# PHLDA1 Modulates the Endoplasmic Reticulum Stress Response and is required for Resistance to Oxidative Stress-induced Cell Death in Human Ovarian Cancer Cells

**DOI:** 10.7150/jca.45262

**Published:** 2021-07-25

**Authors:** Jing Xu, Gang Bi, Qingya Luo, Yi Liu, Tao Liu, Lanfang Li, Qi Zeng, Qien Wang, Yufeng Wang, Jianhua Yu, Ping Yi

**Affiliations:** 1 Department of Obstetrics and Gynecology, The Third Affiliated Hospital of Chongqing Medical University, Chongqing 401120, PR China.; 2 Department of Urology, Daping Hospital, Army Medical University, Chongqing 400042, PR China.; 3 Department of Obstetrics and Gynecology, Daping Hospital, Army Medical University, Chongqing 400042, PR China.; 4 The Ohio State University Comprehensive Cancer Center, Columbus, OH 43210, USA.; 5 Cancer Research Institute of Jilin University, The First Hospital of Jilin University, Changchun 130021, PR China.; 6 Department of Hematology and Hematopoietic Cell Transplantation, Comprehensive Cancer Center, City of Hope National Medical Center, Duarte, California 91010, USA.

**Keywords:** PHLDA1, endoplasmic reticulum stress, oxidative stress, apoptosis, ovarian cancer

## Abstract

**Objective:** Pleckstrin homology-like domain family A member 1 (PHLDA1) has been implicated in the regulation of apoptosis in a variety of normal cell types and cancers. However, its precise pathophysiological functions remain unclear. Here, we examined the expression of PHLDA1 in human ovarian cancer (OvCa), the most lethal gynecologic malignancy, and investigated its functions *in vitro*.

**Materials and Methods:** The expression of PHLDA1 was detected by reverse-transcription quantitative PCR (RT-qPCR), immunohistochemical analysis, or western blotting, silencing of PHLDA was achieved by shRNA, cell proliferation was detected by MTT assay, apoptosis was detected by flow cytometric analysis, PHLDA1 transcriptional activity was detected by dual luciferase reporter assay.

**Results:** PHLDA1 mRNA levels were significantly higher in serous OvCa specimens compared with normal ovarian tissue, confirmed by immunohistochemical staining of PHLDA1 protein, which also indicated the expression was predominantly cytoplasmic. Bioinformatics analysis of publicly available datasets indicated that PHLDA1 expression in clinical specimens was significantly associated with disease stage, progression-free survival, and overall survival. In human OvCa cell lines, shRNA-mediated silencing of PHLDA1 expression enhanced apoptosis after exposure to oxidative stress- and endoplasmic reticulum stress-inducing agents. PHLDA1 silencing increased not the expression of anti-apoptotic or autophagy-related proteins, but the expression of ER stress response-associated proteins.

**Conclusion:** PHLDA1 modulates the susceptibility of human OvCa cells to apoptosis *via* the endoplasmic reticulum stress response pathway.

## Introduction

Epithelial ovarian cancer (OvCa) is the most lethal gynecologic malignancy and the fifth most common cause of cancer-related death in women in the United States 1. High-grade serous ovarian carcinoma (sOvCa) is particularly common and accounts for about 70% of OvCa deaths 2. More than 70% of patients are diagnosed as advanced disease that requires cytoreductive surgery followed by combined platinum and taxane chemotherapy 1,3. The overall 5-year survival rate of patients with advanced OvCa is only 29% 1. Thus, there is a clear need to identify novel molecular biomarkers and therapeutic targets for OvCa.

DNA damage-binding protein 2 (DDB2) is involved in DNA repair and has been shown to suppress the tumorigenicity of OvCa 4. We recently analyzed the transcriptome of a human OvCa cell line stably overexpressing DDB2 and found that they expressed significantly lower level of pleckstrin homology-like domain A family member 1 (PHLDA1) mRNA compared with control cells transfected with the empty vector (~33% of control levels). These results were validated by RT-qPCR analysis and suggested that PHLDA1 might be involved in the tumorigenicity of OvCa 4.

PHLDA1 is a protein containing proline-glutamine and proline-histidine repeat domains 5,6, and was first described to be important in regulating the expression of death receptor CD95/Fas and the activation of apoptotic cell death in a murine T cell hybridoma 5. However, studies with gene-deficient mice have shown that PHLDA1 is not essential for Fas/CD95 regulation and T cell apoptosis in vivo 7. Thus, the physiological role of PHLDA1 in activation-induced apoptosis remains controversial. Expression of PHLDA1 was found to be reduced in primary and metastatic melanomas compared with benign melanocytic nevi, resulted in resistance of the tumor cells to apoptosis 8. PHLDA1 was also shown to play an important role in the anti-apoptotic effects of insulin-like growth factor-1 in breast cancer cells 9. In addition, PHLDA1 contributed to tumor cell proliferation as a putative epithelial stem cell marker in human intestinal tumors 10. Hydrogen sulfide exposure enhanced the expression of PHLDA1 in an oral cancer cell line, Ca9-22, but not in normal oral keratinocytes, and knockdown of PHLDA1 in Ca9-22 cells induced apoptosis 11. In estrogen receptor-positive breast cancer, elevated PHLDA1 expression resulted in enhanced stem-like properties and was associated with a poor clinical outcome 12. Taken together, these reports demonstrated that PHLDA1 might have different functions in the context of different tumor tissues and cell types. While the function of PHLDA1 in OvCa remains unclear.

Here, we examined the expression and functions of PHLDA1 using sOvCa clinical specimens and cell lines. Our results revealed that PHLDA1 was overexpressed in sOvCa specimens and involved in resistance to apoptosis via modulation of the endoplasmic reticulum (ER) stress response. These findings shed light on the functions of PHLDA1 in human OvCa and its role in tumorigenesis.

## Material and Methods

### Cell lines and tissue samples

The human OvCa cell line 2008 was kindly provided by Dr. Francois X. Claret (University of Texas MD Anderson Cancer Center, Houston, TX), and the SKOV3 OvCa cell line was kindly provided by Dr. Thomas C. Hamilton (Fox Chase Cancer Center, Philadelphia, PA). Both cell lines were authenticated by DNA short tandem repeat profiling. The cell lines were maintained in RPMI 1640 medium supplemented with 10% fetal bovine serum, 100 μg/mL streptomycin, and 100 units/mL penicillin, were cultured at 37℃ in a humidified atmosphere of 5% CO_2_ in air. The cell lines were tested as being mycoplasma free by PCR Mycoplasma Test Kit II (AppliChem).

sOvCa tissue and fresh normal ovarian surface epithelium brushings were collected from patients undergoing surgery for sOvCa or benign gynecological diseases in DAPING Hospital between 2009 and 2016. Of these samples, 40 sOvCa specimens and 27 normal brushings obtained between 2013 and 2015 were frozen upon collection, 134 samples of sOvCa and 18 normal ovarian tissues collected between 2009 and 2016 were formalin-fixed and paraffin-embedded upon collection 13. Clinicopathological information, including age, International Federation of Gynecology and Obstetrics (FIGO) 2012 stage, and differentiation were collected from clinical records 13. None of these patients had received chemoradiotherapy before surgery. The study was conducted in accordance with the Code of Ethics of the World Medical Association (Declaration of Helsinki). Informed consent was obtained from all patients, and the study protocol was approved by the Institutional Review Board of DAPING Hospital of Third Military Medical University.

RNA-seq and clinical datasets were downloaded from The Cancer Genome Atlas (http://cbioportal.org) and Kaplan-Meier Plotter (http://kmplot.com).

### RNA isolation and reverse-transcription quantitative PCR (RT-qPCR)

RNA was isolated using TRIzol reagent (Invitrogen, Carlsbad, CA, USA), and then reverse transcribed using Revert Aid First Strand cDNA Synthesis Kit (Thermo Scientific, Waltham, MA, USA) according to the manufacturers' instructions. cDNA was amplified using a 7900HT Fast Real-Time PCR System (Applied Biosystems, Waltham, MA, USA) with SYBR Green Master Mix (New Industry, China). Primers used were: PHLDA1 forward 5′-CCA GGA CAG ATG CTA CTT GG-3′ and reverse 5′-GAC TAC ATA ACC TAG CAG TGG-3′; β-actin forward 5′-CTG GCA CCA CAC CTT CTA CA-3′ and reverse 5′-AGC ACA GCC TGG ATA GCA AC-3′. The reaction conditions were 50 s at 50°C, 5 min at 95°C, followed by 45 cycles of 5 s at 95°C, 15 s at 60°C, and 10 s at 72°C. Relative quantification of mRNA was calculated using the ∆∆Ct method and was normalized with β-actin mRNA. All samples were typically analyzed in triplicate.

### Immunohistochemical analysis (IHC)

Formalin-fixed, paraffin-embedded samples of sOvCa and normal ovarian tissue were fabricated into tissue microarrays. Staining was performed according to standard procedures 13 with a primary anti-PHLDA1 antibody from Abcam (Cambridge, UK; ab47625, 1:150 dilution). Staining of PHLDA1 was scored for both intensity and extent by two investigators independently. Intensity of staining was scored as: negative, 0; weak, 1; moderate, 2; and strong, 3. The percentage of cells with positive staining was scored as: 0%, 0; 1%-25%, 1; 26%-50%, 2; 51%-75%, 3; and >75%, 4. The composite H-score was determined by multiplying the intensity and percentage scores (possible range 0-12) 13.

### PHLDA1 knockdown with short hairpin RNA (shRNA)

2008 and SKOV3 cells were seeded in 6-well plates at 5×10^5^ cells/well one day before transfection. Cells were infected by lentiviral vectors pLKO.1 encoding scrambled shctrl (Addgene, Cambridge, MA, USA) or one of the three PHLDA1-targeting shRNAs (all from Sigma, St. Louis, MO, USA): shPHLDA1-1 (CCG GCC TAA TCC GTA GTA ATT CCT ACT CGA GTA GGA ATT ACT ACG GAT TAG GTT TTTG), shPHLDA1-2 (CCG GCA GAT CAA GTA GTT TGG ACA TCT CGA GAT GTC CAA ACT ACT TGA TCT GTT TTT TG), and shPHLDA1-3 (CCG GCG AGC ACA TTT CTA TTG TCT TCT CGA GAA GAC AAT AGA AAT GTG CTC GTT TTT TG). Stably transfected cells were selected with puromycin and maintained in puromycin-containing medium. PHLDA1 knockdown efficiency was evaluated by western blot analysis.

### MTT proliferation assay

Cell proliferation was assessed using the 3-[4, 5-dimethylthiazol-2-yl]-2, 5 diphenyl tetrazolium bromide (MTT) assay. Cells were seeded in 96-well plates at 10^4^ cells/well in triplicate and incubated for 1-6 days. At the appropriate time, 20 μL/well of CellTiter 96® AQueous One Solution Reagent (Promega, Madison, WI, USA) was added, and the plates were incubated for an additional 1 h. Absorbance at 490 nm was recorded using a 96-well ELISA plate reader.

### Apoptosis assay

Apoptosis was assessed by flow cytometric analysis of cells stained with annexin V-fluorescein isothiocyanate (FITC) and the nucleic acid stain propidium iodide (PI; Invitrogen). Cells were seeded in 6-well plates at 5×10^5^ cells/well overnight, exposed to 0.2 mM H_2_O_2_ or 20 μM thapsigargin (Tg) for 12 h, and then stained with annexin V-FITC and PI. Flow cytometric analysis was performed with a BD LSR II cytometer and FACSDIVA software (both from BD Biosciences, San Jose, CA, USA). A total of 10^4^ events were collected per sample.

### Western blot analysis

Western blotting was performed as previously described (Zhao et al. 2015) with the following primary antibodies: PHLDA1 (MABC753, Millipore, billerica,MA, USA), C-PARP1 (6894S, Cell Signaling Technology, Danvers, MA, USA), C-caspase3 (9664S, Cell Signaling Technology), Beclin-1 (3495, Cell Signaling Technology), P62 (ab56416, Abcam), LC3-I/II (12741, Cell Signaling Technology), Bcl-2 (SC-509, Santa Cruz Biotechnology, Santa Cruz, CA, USA), inositol-requiring protein-1 (IRE1α, 3294, Cell Signaling Technology), protein kinase RNA (PKR)-like ER kinase (PERK, 5683, Cell Signaling Technology), binding immunoglobulin protein (BIP, 3177, Cell Signaling Technology), endoplasmic oxidoreductin-1 (ERO1-Lα, 3264, Cell Signaling Technology), protein disulfide isomerase (PDI, 3501, Cell Signaling Technology), β-actin (sc-1616, Santa Cruz Biotechnology), and Lamin B (sc-6216, Santa Cruz Biotechnology). Bands were visualized using a FluorChem R system (ProteinSimple, Santa Clara, CA, USA).

### Dual luciferase reporter assay

The *PHLDA1* gene promoter sequence from -2148 bp to +239 bp of the transcription start site was amplified from human leukocytes and cloned into the pGL3-basic luciferase reporter vector (Promega) to generate pGL3-PHLDA1. 2008 cells were transfected with either the empty vector or pGL3-PHLDA1 together with pRL-TK Renilla reporter plasmid (Promega) to normalize transfection efficiency. Cells were incubated for 48 h and then treated with 0.2 mM H_2_O_2_ for an additional 4 h. Luciferase activity was then quantified fluorometrically using the Dual Luciferase Assay system (Promega).

### Statistical analysis

The data are expressed as mean ± SD. The Mann-Whitney U test was performed for two-group data, and three-group or four-group data were analyzed using one-way ANOVA. All analyses were conducted using SPSS version 18.0 (SPSS, Chicago, IL, USA). A *P* value less than 0.05 was considered statistically significant.

## Results

### Expression of PHLDA1 was upregulated in serous ovarian cancer compared with normal ovarian surface epithelium

**Figure 1:**
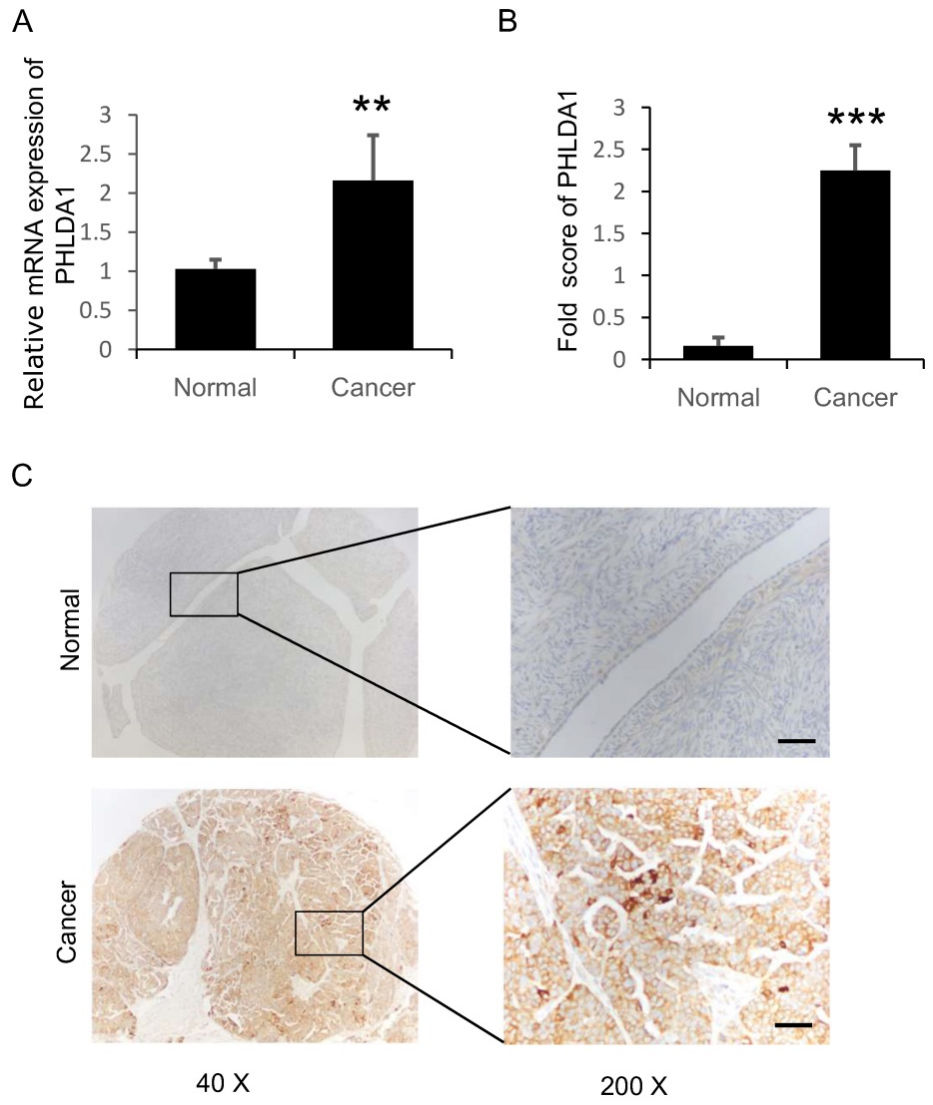
** Expression of PHLDA1 in serous ovarian cancer specimens.** (**A**) RT-qPCR analysis of PHLDA1 mRNA in specimens of sOvCa and normal ovarian surface epithelium. (**B**) Immunohistochemical staining scores for PHLA1 protein expression in sOvCa and normal ovarian tissues. (**C**) Immunohistochemical staining of PHLDA1 detected mainly in the cytoplasm of sOvCa cells (light brown/brown particles), Scale bar, 100 µm. Data are shown as means ± S.D. **P<0.001, *** *P*<0.0001, compared with normal specimens.

PHLDA1 mRNA and protein expression in ovarian tissue samples were assessed using RT-qPCR and IHC analysis, respectively. We performed RT-qPCR analysis on 40 sOvCa and 27 normal specimens and found significantly higher PHLDA1 mRNA levels in the tumor tissues compared with normal tissues (Figure 1A, *P*=0.0007). Three tissue microarrays containing a total of 134 sOvCa and 18 normal ovarian tissues were examined by IHC to localize and quantify PHLDA1 protein expression. Staining was assessed using H-score, a commonly used semi-quantitative method that evaluates both the intensity and extent of protein staining. We found the H score for PHLDA1 expression was high in sOvCa specimens, while low in normal samples (Figure 1B,* P*<0.0001), consistent with the results of the RT-qPCR analysis. Positive PHLDA1 staining was mainly observed in the cytoplasm of sOvCa cells and manifested as light brown or brown particles (Figure 1C). Thus, PHLDA1 mRNA and protein levels were upregulated in sOvCa tissues compared with normal ovarian tissue samples.

### PHLDA1 protected against oxidative stress- and ER stress-induced death in ovarian cancer cells

*In vitro* studies about the roles of PHLDA1 in cancer cell proliferation and survival showed equivocal results, some studies provided evidence for a pro-apoptotic and/or anti-proliferative role 5,8 and others suggested the opposite role 9-12. To assess the role of PHLDA1 on cell growth, we analyzed proliferation of the OvCa cell lines 2008 and SKOV3 expressing shPHLDA1-1, shPHLDA1-2, or shPHLDA1-3 by MTT assay. However, in neither 2008 nor SKOV3 cells line, we could detect difference in proliferation between shctrl and shPHLDA1-expressing groups (data not shown).

**Figure 2:**
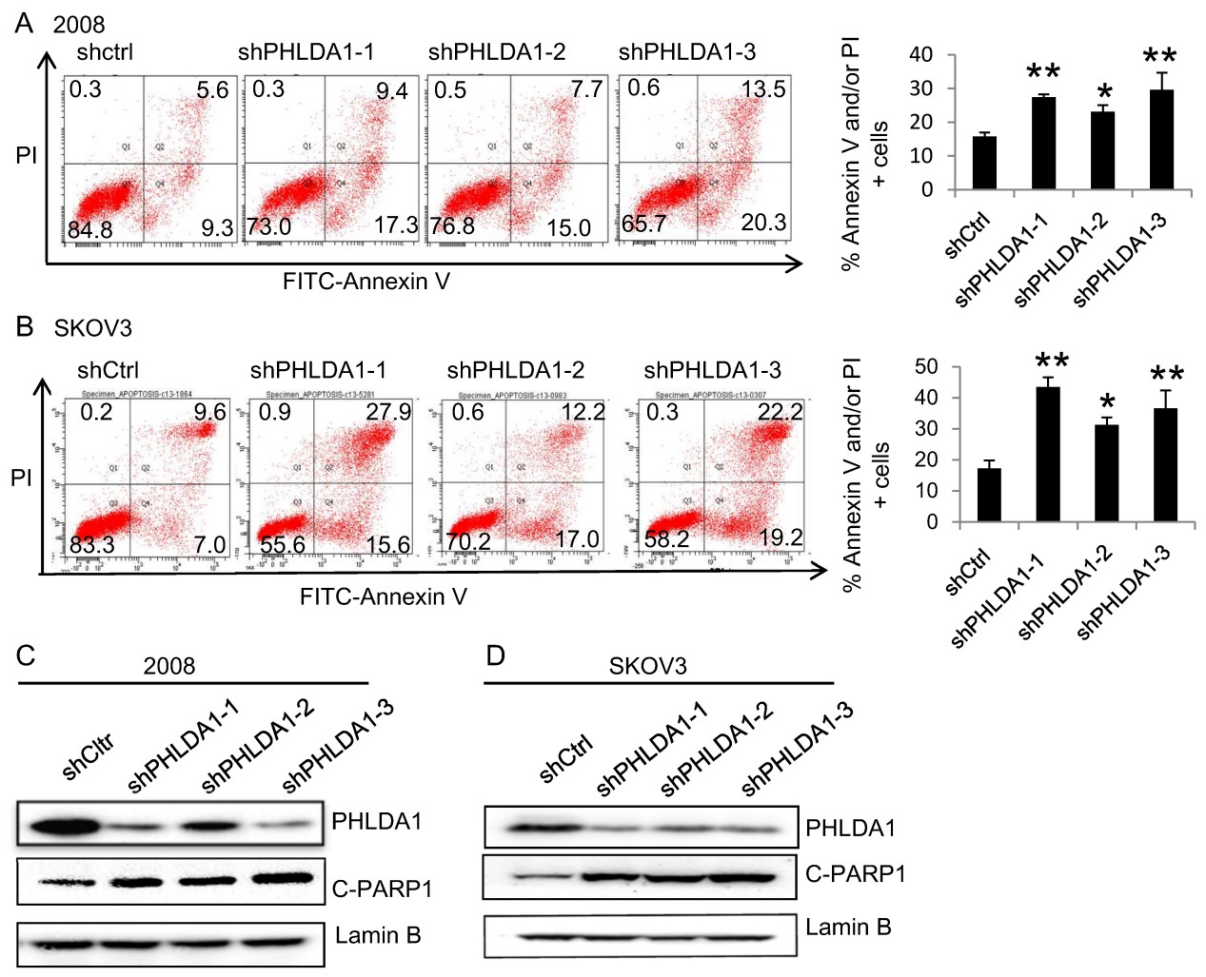
** H_2_O_2_-induced apoptosis of ovarian cancer cells after downregulation of PHLDA1.** (**A and B**) Flow cytometric analysis of 2008 cells (**A**) and SKOV3 cells (**B**) expressing control (shctrl) or PHLDA1-targeting shRNAs (shPHLDA1). Lower right and upper right quadrants showed early apoptotic and late apoptotic/necrotic cells, respectively. n=3, *P<0.05, **P<0.001, compared with the shctrl group. (**C and D**) Western blot analysis of the apoptosis marker c-PARP1 in 2008 (**C**) and SKOV3 (**D**) cell lines expressing shctrl or shPHLDA1.

To evaluate the role of PHLDA1 on OvCa cell death, we examined the effects of shRNA-mediated PHLDA1 downregulation in 2008 and SKOV3. Control (shctrl) and shPHLDA1-expressing cells were incubated with 0.2 mM H_2_O_2_ for 12 h to induce oxidative stress and then stained with annexin V and PI to assess apoptosis. As shown in Figure 2A and B, shPHLDA1, particularly shPHLDA1-1 and shPHLDA1-3, significantly increased the proportion of early apoptosis and late apoptosis/necrosis compared with the shctrl group. Western blot analysis confirmed that PHLDA1 expression was markedly reduced by shPHLDA1; consistent with the flow cytometry results, expression of cleaved poly ADP-ribose polymerase-1 (c-PARP1), a commonly used marker of apoptosis, was increased in the OvCa cells expressing shPHLDA1 compared with the control cells incubated with 0.2 mM H_2_O_2_ for 12 h (Figure 2C and D). Collectively, these results suggested that PHLDA1 suppression significantly increased apoptosis in OvCa cell lines exposed to oxidative stress.

**Figure 3:**
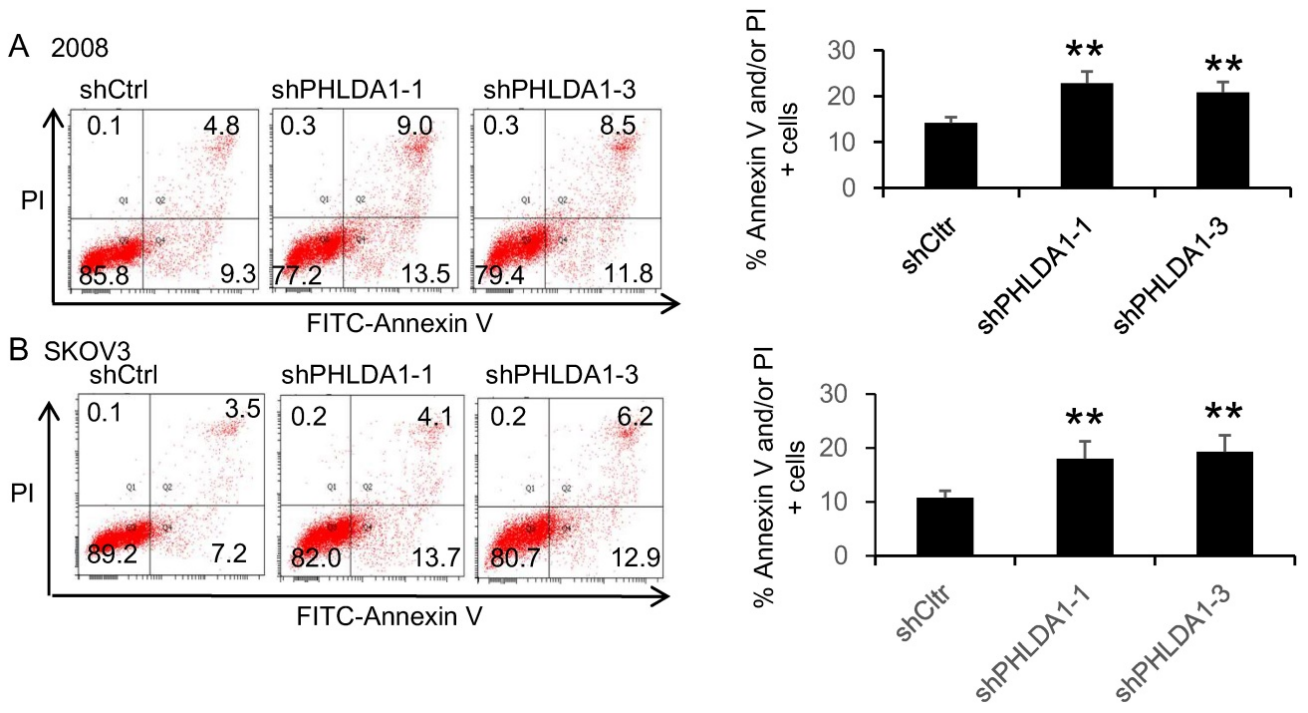
** ER stress-induced apoptosis of ovarian cancer cells after downregulation of PHLDA1**. Apoptosis assay of 2008 (**A**) and SKOV3 (**B**) cells expressing shctrl or shPHLDA1 after treatment with thapsigargin (Tg). **P<0.001 compared with the shctrl group. Mean ± SD, n = 3.

We next evaluated whether PHLDA1 expression could also protect OvCa cells against ER stress. Indeed, incubation with the ER stress inducer 20 μM Tg for 12 h induced a significantly higher rate of apoptosis in 2008 and SKOV3 cells expressing shPHLDA1 compared with shctrl (Figure 3A and B). Taken together, these results indicated that PHLDA1 downregulation increased the susceptibility of OvCa cells to oxidative stress- and ER stress-induced apoptosis, but does not affect cell proliferation.

### Oxidative stress directly increased PHLDA1 transcription in ovarian cancer cells

**Figure 4:**
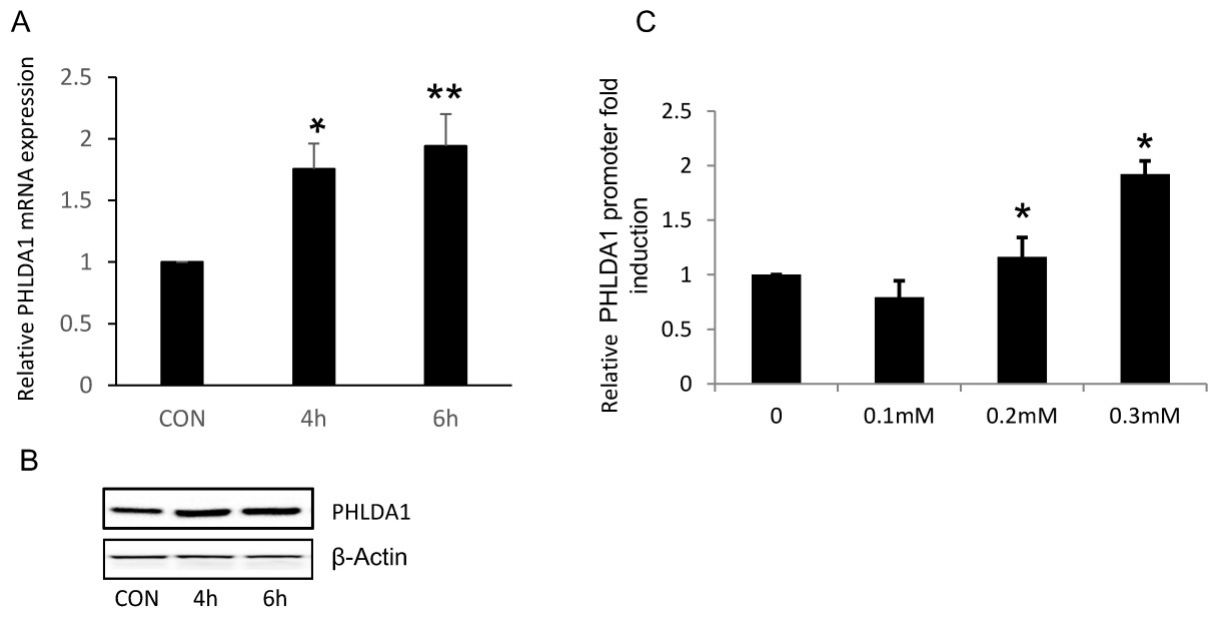
** H_2_O_2_-induced changes of PHLDA1 expression in ovarian cancer cells**. (**A**) RT-qPCR analysis of PHLDA1 mRNA levels in 2008 cells treated with H_2_O_2_. (B) Western blot analysis of PHLDA1 in 2008 cells treated with H_2_O_2_. (**C**) Luciferase assay of 2008 cells transfected with a luciferase vector driven by the PHLDA1 promoter after treatment with H_2_O_2_. n=3, *P<0.05, **P<0.001, compared with the control group.

Because our results suggested that PHLDA1 might play a protective role against oxidative stress- and ER stress-induced apoptosis in OvCa cells, we next determined whether exposure to oxidative stress directly increased PHLDA1 expression, as has been previously demonstrated in mouse embryonic fibroblasts 14. Indeed, RT-qPCR analysis revealed that PHLDA1 mRNA levels were enhanced in 2008 cells treated with 0.2 mM H_2_O_2_ for 4 h or 6 h (Figure 4A). Western blot analysis confirmed that PHLDA1 expression was enhanced in 2008 cells treated with H_2_O_2_ (Figure 4B). To further examine the mechanism of PHLDA1 mRNA upregulation induced by oxidative stress, we generated a luciferase reporter plasmid (pGL3-PHLDA1) harboring ~2.4 kb of the human *PHLDA1* promoter sequence upstream of the luciferase gene. 2008 cells were transfected with empty vector or pGL3-PHLDA1 for 48 h and then treated with 0.1-0.3 mM H_2_O_2_ for 4 h before analysis of luciferase secretion. The results showed that exposure to H_2_O_2_ dose-dependently increased luciferase secretion in pGL3-PHLDA1-expressing 2008 cells (Figure 4C). Which suggested that exposure to oxidative stress induced PHLDA1 transcription and upregulated PHLDA1 mRNA in OvCa cells.

### PHLDA1 modulated the endoplasmic reticulum stress response in ovarian cancer cells

**Figure 5:**
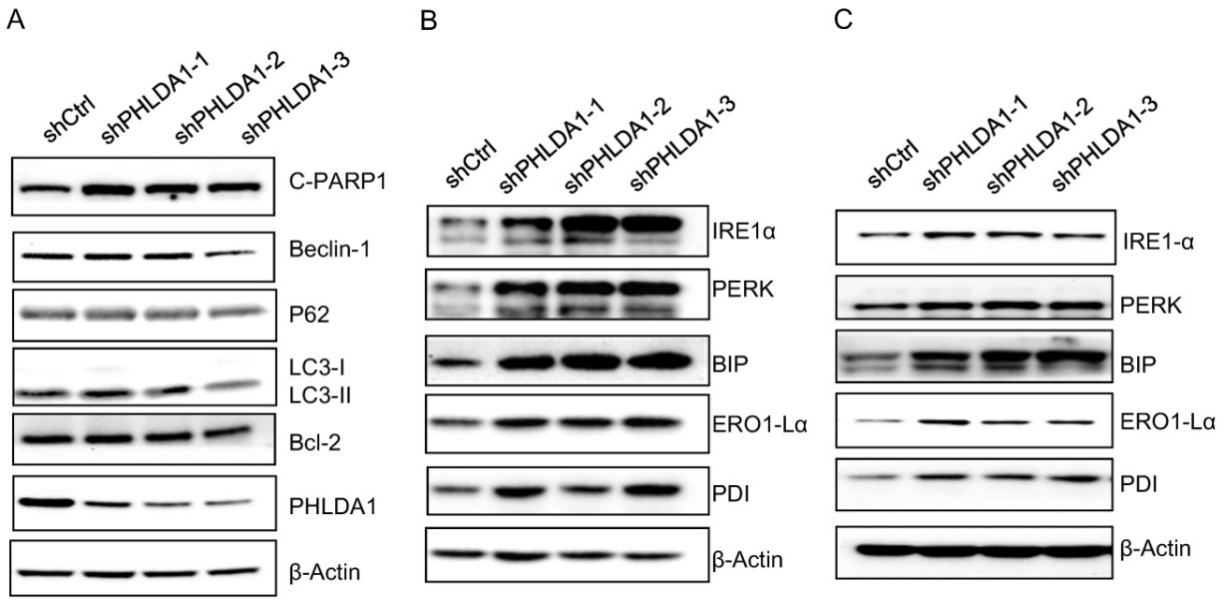
** Expression of autophagy-related proteins, anti-apoptosis proteins, ER stress-associated proteins after PHLDA1-downregulation in ovarian cancer cells.** (**A**) Western blot analysis of Autophagy-related proteins (Beclin-1, P62, and LC3) and anti-apoptosis protein (Bcl-2) in 2008 cells expressing shctrl or shPHLDA1 after treatment with 0.2 mM H_2_O_2_. (**B and C**) Western blot analysis of ER stress-associated proteins (IRE1α, PERK, BIP, ERO1-Lα, and PDI) in 2008 (**B**) and SKOV3 (**C**) cells after treatment with 0.2 mM H_2_O_2_.

Our results are consistent with several other studies indicating downregulation of PHLDA1 could promote oxidative stress-induced cell death 9,11,14; however, the mechanism is not yet fully understood. To determine whether PHLDA1-relative cell death might be caused by inhibition of autophagy or anti-apoptotic pathways, we examined the expression of the autophagy-related proteins (Beclin-1, P62, and LC3), and the anti-apoptotic protein Bcl-2 by western blotting. The results showed shPHLDA1-mediated knockdown did not alter the expression of these proteins in 2008 cells exposed to H_2_O_2_ (Figure 5A).

Given that PHLDA1 silencing promoted the apoptosis of 2008 and SKOV3 cells induced by the ER stress inducer Tg (showed in Figure 3), we also examined the expression of the ER stress-responsive proteins IRE1α, PERK, BIP, ERO1-Lα, and PDI. Indeed, both in 2008 cells (Figure 5B) and SKOV3 cells (Figure 5C), the expression of all of these proteins was found increased by H_2_O_2_ treatment to a greater extent in shPHLDA1-expressing cells compared with control cells. Given that ER stress could induce cell death 15, these results suggested that PHLDA1 might protect against oxidative stress-induced apoptosis by modulating the expression of proteins involved in the ER stress response.

## Discussion

Apoptosis serves as a natural barrier to cancer development, and resistance to apoptosis is a hallmark of malignant cells 16. Therefore, understanding the mechanisms by which cancer cells evade apoptosis is crucial for the development of novel therapies.

PHLDA1 was first identified as a modulator of T cell apoptosis more than 20 years ago, and since then, there were several reports revealed that PHLDA1 might have both pro- and anti-apoptotic functions depending on the cells and tumor context 6-11,17. We showed here that PHLDA1 was upregulated in sOvCa compared with normal specimens and played an important role in cell apoptosis, as measured by annexin V-FITC/PI staining and expression of c-PARP1, but not in cell proliferation, as measured by the MTT assay. The anti-apoptotic function of PHLDA1 suggests that it may act as an oncogene in human sOvCa. Bioinformatic analysis of publicly available OvCa datasets (http://kmplot.com and http://cbioportal.org) indicated that low level of PHLDA1 conferred a survival benefit to OvCa patients, consistent with our *in vitro* findings. Thus, PHLDA1 could be a potential therapeutic target and/or prognostic marker for OvCa.

Accumulation of reactive oxygen species (ROS), including H_2_O_2_, can activate multiple cellular signaling pathways and promote cancer development 18. Baseline ROS level has been shown to be much higher in OvCa cells than an immortalized ovarian epithelial cell line 19,20. However, excessive ROS can also induce pro-apoptotic pathways, leading to cell death 21,22; as such, resistance to apoptosis induced by oxidative stress is crucial for cancer cell survival and is involved in the development of chemoresistance in many cancers 23. We found that H_2_O_2_ treatment upregulated PHLDA1 expression by promoting its transcription, and downregulation of PHLDA1 promoted oxidative stress-induced apoptosis, which revealed that PHLDA1 played a protective role in OvCa cells. Notably, knockdown of PHLDA1 did not increase the expression of the key autophagy-associated proteins (Beclin-1, P62, and LC3), or the anti-apoptotic protein Bcl-2 in response to H_2_O_2_ treatment, but did increase the expression of the ER stress-associated proteins IRE1α, PERK, BIP, ERO1-Lα, and PDI. We also showed that PHLDA1 downregulation enhanced apoptosis in response to the ER stress inducer Tg. Given that ER stress can induce excessive ROS production and cell death 15, 24, our results suggested that low PHLDA1 expression might promote apoptosis of OvCa cells induced by ER stress. In support of this, PHLDA1 has previously been implicated in regulation of the ER stress response in other cell types 14, 25.

## Conclusions

PHLDA1 expression is enhanced in sOvCa compared with normal tissue and is significantly associated with FIGO stage. PHLDA1 appears to play an important role in apoptosis resistance in OvCa cell lines through a mechanism involving the ER stress response. PHLDA1 may be a novel prognostic biomarker and a therapeutic target for OvCa. Further studies are required to explore the regulatory mechanism by which PHLDA1 modulates apoptosis upon cell exposure to ER stress and oxidative stress.

## Abbreviations

PHLDA1: Pleckstrin homology-like domain family A member 1
OvCa: ovarian cancer
sOvCa: serous ovarian carcinoma
ER: endoplasmic reticulum
DDB2: DNA damage-binding protein 2
IHC: Immunohistochemical analysis
shRNA: short hairpin RNA
